# MID-LIFE JOB STRESS AND SUPPORT PREDICT SLEEP HEALTH TRAJECTORIES THROUGHOUT ADULTHOOD

**DOI:** 10.1093/geroni/igac059.582

**Published:** 2022-12-20

**Authors:** Soomi Lee, Claire Smith, Tammy Allen, Meredith Wallace, David Almeida, Orfeu Buxton, Sanjay Patel, Ross Andel

**Affiliations:** University of South Florida, Tampa, Florida, United States; University of South Florida, Tampa, Florida, United States; University of South Florida, Tampa, Florida, United States; University of Pittsburgh, Pittsburg, Pennsylvania, United States; The Pennsylvania State University, University Park, Pennsylvania, United States; The Pennsylvania State University, University Park, Pennsylvania, United States; University of Pittsburgh, Pittsburgh, Pennsylvania, United States; University of South Florida, Tampa, Florida, United States

## Abstract

Good sleep is necessary for healthy aging, but it may be threatened by work stress. This study connected midlife job characteristics to trajectories of sleep health profiles (within-person configurations of key self-reported facets: duration, regularity, sleep onset latency or SOL, insomnia symptoms, feeling unrested, and napping) over one decade. A working adult sample (N=847, Mage=45) of the Midlife in the United States study provided data on sleep and job characteristics in 2004-2006 (T1) and 2013-2016 (T2). Four sleep profiles were consistently identified at both time points: (1) good sleepers, (2) irregular but sufficient, (3) short sleepers, and (4) long SOL/insomnia. Higher job demands at T1 predicted a transition from good or irregular/sufficient sleep at T1 to long SOL/insomnia at T2. Higher workplace social support at T1 predicted maintenance of good or irregular/sufficient sleep over time. Attention to job demands and workplace social support may help promote sleep health.

